# Maternal plasma choline and betaine in late pregnancy and child growth up to age 8 years in the KOALA Birth Cohort Study

**DOI:** 10.1093/ajcn/nqab177

**Published:** 2021-06-10

**Authors:** Carolina Moltó-Puigmartí, Rima Obeid, Monique Mommers, Simone Jpm Eussen, Carel Thijs

**Affiliations:** Department of Epidemiology, Maastricht University Medical Centre^+^, Maastricht, Netherlands; Department of Clinical Chemistry and Laboratory Medicine, Saarland University Hospital, Homburg, Germany; Department of Epidemiology, Maastricht University Medical Centre^+^, Maastricht, Netherlands; Department of Epidemiology, Maastricht University Medical Centre^+^, Maastricht, Netherlands; Care and Public Health Research Institute, CARIM School for Cardiovascular Diseases, Maastricht University, Maastricht, Netherlands; Department of Epidemiology, Maastricht University Medical Centre^+^, Maastricht, Netherlands

**Keywords:** betaine, choline, pregnancy, one-carbon metabolism, birth weight, infant growth, overweight, fetal programming

## Abstract

**Background:**

Sufficient choline and betaine during pregnancy are needed for fetal growth and development.

**Objectives:**

We aimed to investigate the associations between maternal plasma choline and betaine in the third trimester of pregnancy and child growth from birth up to 8 years of age.

**Methods:**

Concentrations of choline and betaine were measured in plasma of 1331 pregnant women from the KOALA (Kind, Ouders en gezondheid: Aandacht voor Leefstijl en Aanleg) Birth Cohort Study in the Netherlands. Child weight and height were measured at birth and at 1 (91% complete), 2 (86%), and 6–8 y (76%). Birth weight, weight gain in the first year, and *z* scores for weight and height at 1 and 2 y were used as continuous outcome variables. BMI *z* scores at 1 and 2 y were used as continuous and dichotomous outcomes, and BMI *z* scores at age 6–8 y were used to study overweight at that age.

**Results:**

Each 1-µmol/L increase of maternal plasma choline was associated with a mean 20-g (95% CI: 1.1, 38.0 g) higher weight gain in the first year of life, and a higher BMI *z* score (β: 0.02; 95% CI: 0.00, 0.04) and slightly higher odds of BMI *z* score >85th percentile (OR: 1.08; 95% CI: 1.03, 1.10) at 1–2 y. Each 1-µmol/L increase of plasma betaine was associated with a mean 12-g (95% CI: 0.8, 23.9 g) higher weight gain in the first year of life and higher odds of BMI *z* score >85th percentile at 1–2 y (OR: 1.03; 95% CI: 1.00, 1.07). Lastly, betaine was associated with overweight at 6–8 y (OR: 1.17; 95% CI: 1.02, 1.34), only in boys.

**Conclusions:**

Third-trimester pregnancy plasma choline and betaine were positively associated with childhood anthropometric measures. In boys, some of the associations may have persisted up to 8 y of age. Further studies may investigate the validity of maternal plasma choline and betaine concentrations as markers of maternal intake and fetal transfer.

## Introduction

Choline is an essential nutrient for humans (1). It is partly synthesized in the liver, but must also be obtained through the diet (2, 3). Choline is a source of acetylcholine (neurotransmitter) and phosphatidylcholine, the latter being an integral part of cell membranes and lipoprotein-transporting particles in blood (4, 5). Besides, choline and its oxidation product betaine, as well as folate, are involved in one-carbon metabolism and epigenetic regulation of key genes, thereby participating in the phenomenon of fetal programming (6). Supplementing choline to the mother during pregnancy may influence functional processes in the placenta such as improving angiogenesis and nutrient transport, and reducing the impact of inflammation processes (7). During pregnancy, the requirements for choline increase (8) to ensure the fetal supply (9). The hormonal changes occurring in pregnancy contribute to increasing concentrations of choline; besides, choline is actively transferred to the fetus (10) and reaches higher concentrations in cord blood than in maternal blood (11–13).

In animal models for maternal obesity and gestational diabetes, choline influenced fetal adiposity, insulin resistance, and placental response to hyperglycemia (14, 15). Similarly, maternal betaine supplementation influenced fetal growth and lipid metabolism, possibly through both shared and unique mechanisms (16). In humans, the evidence of an association of maternal prenatal choline and betaine concentrations with offspring anthropometric measures at birth is limited and conflicting. Moreover, prospective studies on the association of plasma choline and betaine with infant growth beyond birth are scarce (17). Plasma choline concentrations in pregnancy were found to be unrelated to birth weight (12, 18, 19), whereas 1 study with almost 2000 women reported a positive association between maternal plasma choline concentrations during pregnancy and offspring BMI, skinfold thickness, and adiposity at birth, but not with growth and adiposity up to age 5 y (17). Maternal plasma betaine concentrations, on the other hand, were found to be associated with lower birth weight (19, 20) and length, smaller midupper arm circumference, reduced subcutaneous adipose tissue, and a higher risk of being born small for gestational age (20).

In the current study we investigated whether maternal plasma concentrations of both choline and betaine in the third trimester of pregnancy were associated with anthropometric measures of the child at birth and at 1 and 2 y of age, and with overweight at 6–8 y of age.

## Methods

### Study design

This research was performed within the KOALA (Kind, Ouders en gezondheid: Aandacht voor Leefstijl en Aanleg) Birth Cohort Study in the Netherlands, whose general study design has been described in detail previously (21). KOALA is an acronym in Dutch for “child, parents and health: lifestyle and genetic constitution.” Between 2000 and 2002, healthy pregnant women who had been recruited at 14–16 weeks of gestation in the context of another prospective study (Pregnancy-related Pelvic Girdle Pain Study) were invited, at 34 weeks of gestation, to participate with their child in the KOALA study. Most women recruited by this means had a conventional lifestyle in terms of diet and child-rearing practices (conventional recruitment group). In order to enrich the cohort with women with alternative lifestyles, between 2001 and 2002 additional recruitment channels were used: organic food shops, anthroposophical doctors and midwives, and Steiner schools (alternative recruitment group). In total, 2993 pregnant women were recruited (*n* = 2487 in the conventional recruitment group, *n* = 506 in the alternative recruitment group). From October 2001, coinciding with the start of the recruitment of the alternative group, the pregnant women were also asked to give informed consent for blood collection. The women and their children were followed up during pregnancy and up to 11 y after delivery. The study was approved by the Ethics Committee of the Maastricht University/University Hospital Maastricht. All parents gave written informed consent.

### Participants

**FIGURE 1:**
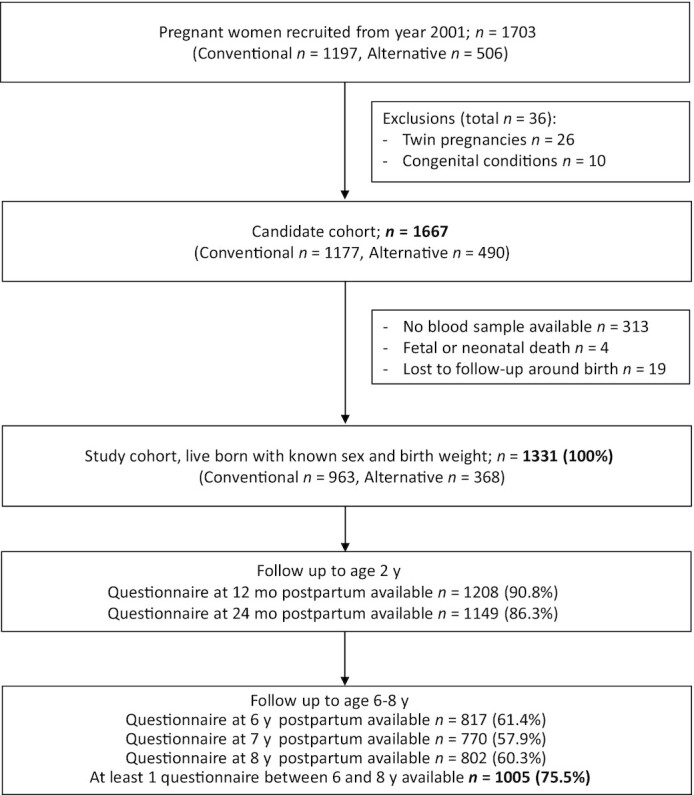
Study flowchart.

The present study is confined to those participants who were recruited from the year 2001 onwards, and therefore were asked to provide consent for blood collection (*n* = 1703: *n* = 1197 from the conventional and *n* = 506 from the alternative recruitment group). Exclusion criteria for the present study were applied in 2 steps: first, multiple pregnancies and newborns with congenital diseases or diseases affecting growth (e.g., Down syndrome) were excluded, leading to the “candidate cohort” (see **Figure 1**). Second, cases were removed where no blood sampling occurred, birth weight information was unavailable, or there was no response to any of the questionnaires in the first year of life, leading to the “birth cohort.” In total, 1331 mother–infant pairs were available for the final analyses (*n* = 963 and *n* = 368 from the conventional and alternative recruitment groups, respectively) (Figure 1).

### Blood collection and biomarker measurements

A 10-mL maternal blood sample was collected into EDTA-K+-containing tubes between 34 and 36 weeks of gestation. Collection took place during home visits conducted by a trained study nurse. Within 30 min of collection, blood samples were centrifuged on-site at 3000 rpm for 10 min at room temperature. The plasma was transferred into cryogenic vials, transported at 4°C to the laboratory, and stored at −80°C in the biobank until further analysis.

Betaine and free choline were measured in EDTA plasma samples by Acquity Ultra Performance Liquid Chromatography coupled to a MicroMass Quattro Premier XE tandem quadrupole mass spectrometer (UPLC-MS/MS, Waters Corporation) as described in Kirsch et al. (22). Briefly, 100 µL blood from each participant was used. Acetonitrile (300 µL) was added to each sample to precipitate the proteins and, after mixing on a vortex and centrifuging at 3000 rpm for 10 min at room temperature, the supernatant was transferred to glass vials and immediately measured by UPLC-MS/MS. Isotope-labeled d9-betaine chloride and d9-choline chloride (Isotec, Sigma-Aldrich) were used as internal standards. The KOALA samples were analyzed in 27 independent runs over 16 d. Quality control samples (with low and high known concentrations of choline and betaine) were analyzed every day together with the study samples and were used to calculate the between-day CVs. The between-day CVs for choline were 3.04% at 4.5 µmol/L and 2.86% at 45 µmol/L. The CVs for betaine were 2.02% at 7.5 µmol/L and 1.56% at 75 µmol/L.

### Maternal covariates

The mothers filled out questionnaires at 14 and 34 weeks of pregnancy, yielding data on maternal characteristics such as height and weight before pregnancy, gravidity, age, highest achieved educational level, living region within the Netherlands, and country of origin of mother's and father's parents, as well as about smoking and alcohol consumption, and use of supplements containing folic acid.

### Child growth data

Birth weight as well as pregnancy duration were reported by the mothers at 2 wk postpartum and were verified against midwives’ records. At ages 1 and 2 y, parents were asked to report the most recent weight and height measured at the child health clinic, and to report the children's age (mo) at the time of these measurements (23). In 3 questionnaires filled out around ages 6, 7, and 8 y, parents were asked to measure their children's weight (in kilograms to 1 decimal place) and height (cm), with the child wearing light clothes and no shoes, and to report the exact date of measurement. In a subgroup of 648 children at ages between 6 and 8 y, weight and height were measured during home visits by trained research assistants, with the children wearing only their underpants. Height was measured with a portable stadiometer (Leicester height measure) and weight was measured with a digital scale (HE-5, CAS Corp.) and recorded in millimeters and grams (rounded off to the nearest 100 g), respectively (23).

BMI was calculated as kg/m^2^. In order to standardize children's weight, height, and BMI for gender and actual age of measurement (which could slightly differ among children), measurements were converted to SD scores (*z* scores) using data from the Dutch reference population as the standard (24).

### Statistical analyses

Statistical analyses were performed using the software package SPSS Statistics version 25 for Windows (IBM Corp.). *P* values < 0.05 were considered statistically significant. All listed anthropometric measurements were considered primary outcome variables that together describe early growth.

Potential influential outliers were evaluated using Cook's distance. Deviation from linearity of the relations of choline and betaine with the studied outcomes was evaluated by quadratic and cubic curve fit (separately for boys and girls); all terms were nonsignificant and hence we proceeded with linear models. Birth weight, weight gain in the first year, and *z* scores for weight and height at the ages of 1 and 2 y were used as continuous outcome variables. BMI *z* scores at 1 and 2 y were used as both continuous and dichotomous outcomes: BMI *z* scores were dichotomized as <−1.04 and >1.04 (corresponding to the 15th and the 85th percentile, respectively) (23). Overweight (yes/no) at 6–8 y was used as a dichotomous outcome with the cutoff at BMI *z* score >1.04 (corresponding with the 85th percentile) (23).

We used multivariable linear regression to analyze the associations of maternal plasma choline and betaine concentrations with birth weight and weight gain in the first year after birth. To analyze the longitudinal association of maternal plasma choline and betaine concentrations with *z* scores of child weight, length, and BMI at 1 and 2 y we used generalized estimation equations (GEEs) for repeated measurements with an unstructured correlation structure. Logistic GEEs were used to study the associations with the odds of having a BMI *z* score <−1.04 or >1.04 at 1–2 y of age and with overweight at age 6–8 y. In all GEE models, the age of the child at measurement (d) of the growth outcome was adjusted for, and time-interactions were tested to evaluate whether the results differed by age of the child at measurement (in separate analyses for ages 1 and 2 y, and for the repeated measurements around ages 6, 7, and 8 y); none of these interactions were statistically significant at α = 0.05 (2-sided), and hence the results are presented for ages 6, 7, and 8 y combined (and expressed as 6–8 y). For the outcome of overweight based on measurements done at home visits (a single measurement between 6 and 8 y), we used logistic regression analysis, adjusting for the same covariates as the GEE analyses.

Potential confounders were selected based on a priori knowledge. For each of the outcomes, several models were tested with and without additional covariates as summarized in **Supplemental Table 1**. We started with a model (step 1) that included child variables (i.e., sex, exact age at outcome measurement—except for birth weight—to account for differences in the age of measurement) and design variables (i.e., sample storage time, type of tube used for storage, pregnancy duration at the time of blood collection, year and season of blood collection, and recruitment group). In step 2 we added ethnicity of the child (based on the country of origin of their grandparents), living region within the Netherlands, maternal education, gravidity, maternal age at the beginning of pregnancy, maternal height, prepregnancy BMI, and alcohol use and smoking in the third trimester. In step 3 we added gestational diabetes as a covariate. We did not adjust for gestational age at birth and birth weight because they are potential mediators and should therefore not be regarded as confounders. Missing values in maternal height for 17 participants were imputed using information on maternal ethnicity, to ensure that the numbers of subjects were identical in each pair of uni- and multivariable models. All results are expressed as regression coefficients (βs) or ORs and 95% CIs per 1 µmol/L choline or betaine and, in the results tables and in order to aid the interpretation, also as effect sizes for an increment of 1 SD of choline or betaine.

Finally, several sensitivity analyses were performed (step 4), including the exclusion of women delivering prematurely (1% of the women) or those with gestational diabetes (1.5%), and effect modification by child's sex, breastfeeding duration (assuming that a longer breastfeeding period would imply an extended duration of exposure to maternal choline and betaine via breast milk), and folic acid supplementation in pregnancy [given that folate and choline are competitive methyl donors, i.e., diminished folate availability increases the demand for choline and vice versa (25)]. Effect modification was evaluated by testing for statistical interaction between these variables and choline or betaine, respectively, at α = 0.05. Folic acid supplementation was categorized as “never supplemented,” “continued use throughout the pregnancy,” and “discontinued use” (i.e., started before or in the first trimester, but stopped before or in the second trimester); effect modification was evaluated by testing interaction terms with the 2 dummy variables for these 3 categories in the same model.

To evaluate selective loss to follow-up, maternal baseline characteristics, birth outcomes, children's anthropometric measures, and choline and betaine concentrations were compared between the participants with complete follow-up and those from the candidate and birth cohorts (see Figure 1 and the Results).

## Results

### Maternal baseline characteristics

**Table 1** presents maternal baseline characteristics. No substantial differences were observed between the candidate cohort, the birth cohort, and the subjects with complete follow-up (see Figure 1 for the different subcohorts), suggesting no selective attrition that could have led to selection bias.

**TABLE 1:** Baseline characteristics of the participant women from the candidate cohort, the birth cohort, and those with complete follow-up^1^

	Candidate cohort (*n* = 1667)	Birth cohort, live-born children with known sex and birth weight (*n* = 1331)	Complete follow-up^2^ (*n* = 1005)
Age of the mother at the start of pregnancy,^3^ y	31.7 ± 3.9	31.8 ± 3.8	31.9 ± 3.8
<25^4^	72 (4.3)	49 (3.7)	37 (3.7)
25–37.99^4^	1493 (89.8)	1204 (90.5)	905 (90.0)
≥38^4^	98 (5.9)	78 (5.9)	63 (6.3)
Missing, *n*	4	—	—
Prepregnancy BMI,^3^ kg/m^2^	23.4 ± 3.8	23.4 ± 3.7	23.4 ± 3.6
<18.5^4^	48 (3.1)	40 (3.0)	26 (2.6)
18.5–24.99^4^	1108 (71.8)	948 (71.5)	720 (71.9)
25–29.99^4^	279 (18.1)	246 (18.6)	193 (19.3)
≥30^4^	108 (7.0)	91 (6.9)	62 (6.2)
Missing, *n*	124	6	4
Maternal height, m	1.7 ± 0.1	1.7 ± 0.1	1.7 ± 0.1
Maternal region of residence within the Netherlands			
South	495 (29.7)	437 (32.8)	324 (32.2)
Other	1172 (70.3)	894 (67.2)	681 (67.8)
Maternal education			
University	282 (16.9)	225 (16.9)	187 (18.6)
Higher vocational	605 (36.3)	498 (37.4)	384 (38.2)
Mid	581 (34.9)	467 (35.1)	339 (33.7)
Lower	134 (8.0)	99 (7.4)	68 (6.8)
Other/unknown	65 (3.9)	42 (3.2)	27 (2.7)
Maternal smoking in the third trimester of pregnancy, cigarettes/d			
0^4^	1533 (95.1)	1264 (95.0)	970 (96.5)
1–9^4^	42 (2.6)	38 (2.9)	22 (2.2)
≥10^4^	37 (2.3)	29 (2.2)	13 (1.3)
Missing, *n*	55	—	—
Alcohol intake in the third trimester of pregnancy, glasses/wk			
0^4^	1325 (82.2)	1079 (81.1)	822 (81.9)
1–2^4^	231 (14.3)	205 (15.4)	151 (15.0)
≥2^4^	55 (3.4)	46 (3.5)	31 (3.1)
Missing, *n*	56	1	1
Diabetes in pregnancy	28 (1.7)	20 (1.5)	17 (1.7)
Maternal gravidity			
1	661 (39.7)	520 (39.1)	402 (40.0)
2	592 (35.5)	462 (34.7)	364 (36.2)
≥3	414 (24.8)	349 (26.2)	239 (23.8)

1 Values are mean ± SD or *n* (%) unless otherwise indicated.

2 Complete follow-up: ≥1 outcome measurement between ages 6 and 8 y.

3 Missings excluded.

4 Percentage of valid values (i.e., excluding missing values).

Women in the birth cohort had a mean age of 31.8 y at the inception of pregnancy. Seventy-two percent of them had a prepregnancy BMI between 18.5 and 25. Sixty percent of the women were multigravida, 1.5% had diabetes in pregnancy, and some reported having smoked (5%) or drunk alcohol (19%) in the third trimester of pregnancy. Just over half had higher education (higher vocational or university).

### Maternal choline and betaine, birth weight, and child anthropometric measures

Mean ± SD maternal plasma choline and betaine concentrations were 11.3 ± 2.9 µmol/L and 11.5 ± 4.6 µmol/L, respectively. The mean ± SD birth weight was 3560 ± 477 g. The percentage of children born with a birth weight <2500 g was 1.3%, whereas 3.3% were born with a birth weight ≥4500 g (macrosomia). Less than 3% of the children were not of European descent. As **Table 2** shows, these figures were comparable with those in the group of participants with complete follow-up. The same was found for the children's anthropometric data at ages 1 and 2 y (Table 2). The mean ± SD ages of the children when weight and height were actually measured were 11.4 ± 0.8 mo at the 1-y measurement, 22.5 ± 3.4 mo at the 2-y measurement, and 6.1 ± 0.4, 6.8 ± 0.4, and 8.7 ± 0.4 y at the 6-, 7-, and 8-y measurements, respectively.

**TABLE 2:** Maternal plasma concentrations of choline and betaine, birth outcomes, and child anthropometric data up to age 6–8 y for the birth cohort and participants with complete follow-up^1^

	Birth cohort, live-born children with known sex and birth weight (*n* = 1331)	Mothers with complete follow-up^2^ (*n* = 1005)
Plasma choline, µmol/L	11.3 ± 2.9	11.4 ± 3.0
Plasma betaine, µmol/L	11.5 ± 4.6	11.6 ± 4.6
Pregnancy duration at the time of blood collection, wk	36.7 ± 1.2	36.7 ± 1.2
<36	347 (26.1)	280 (27.9)
36–37	472 (35.5)	345 (34.3)
>37	512 (38.5)	380 (37.8)
Season of blood collection		
Spring	262 (19.7)	199 (19.8)
Summer	300 (22.5)	231 (23.0)
Autumn	339 (25.5)	268 (26.7)
Winter	430 (32.3)	307 (30.5)
Storage time between blood collection and laboratory measurements, y	17.3 ± 0.4	17.3 ± 0.4
Gestational age at birth, wk	39.7 ± 1.2	39.7 ± 1.2
34–37	13 (1.0)	12 (1.2)
>37	1318 (99.0)	993 (98.8)
Child sex male	679 (51.0)	516 (51.3)
Child ethnicity estimated as grandparents born in Europe, *n*		
3 or 4	1294 (97.2)	981 (97.6)
<3 or unknown	37 (2.8)	24 (2.4)
Birth weight, g	3560 ± 477	3565 ± 476
<2500	17 (1.3)	15 (1.5)
2500–2999	117 (8.8)	83 (8.3)
3000–3999	969 (72.8)	739 (73.5)
4000–4499	184 (13.8)	133 (13.2)
≥4500	44 (3.3)	35 (3.5)
Breastfeeding duration,^3^ mo	5.7 ± 4.4	6.0 ± 4.4
0^4^	171 (12.9)	114 (11.3)
0–3^4^	380 (28.8)	277 (27.6)
4–6^4^	249 (18.8)	201 (20.0)
7–12^4^	358 (27.1)	283 (28.2)
>12^4^	163 (12.3)	130 (12.9)
Missing, *n*	10	—
Child growth data at age 1 y		
*n*	1208	963
Age at measurement, mo	11.4 ± 0.8	11.4 ± 0.8
Weight in kg; weight *z* score	9.6 ± 1.1; −0.1 ± 0.9	9.6 ± 1.1; −0.1 ± 1.0
Height in cm; height *z* score	75.0 ± 2.9; −0.0 ± 1.0	75.0 ± 3.0; −0.1 ± 1.0
BMI in kg/m^2^; BMI *z* score	17.1 ± 1.4; −0.1 ± 1.0	17.1 ± 1.3; −0.1 ± 1.0
Child growth data at age 2 y		
*n*	1149	939
Age at measurement, mo	22.5 ± 3.4	22.4 ± 3.4
Weight in kg; weight *z* score	12.2 ± 1.5; −0.1 ± 1.0	12.2 ± 1.5; −0.1 ± 1.0
Height in cm; height *z* score	86.3 ± 4.5; −0.1 ± 1.1	86.2 ± 4.6; −0.1 ± 1.0
BMI in kg/m^2^; BMI *z* score	16.4 ± 1.4; −0.0 ± 1.0	16.4 ± 1.4; −0.0 ± 1.0
Child growth data at age 6–8 y		
*n*		817; 770; 802
Age at measurement, y		6.1 ± 0.4; 6.8 ± 0.4; 8.7 ± 0.4
Children with ≥1 measurement between age 6 and 8 y, *n*		1005
Children with 1, 2, and 3 measurements, *n*		172, 282, 551
Overweight children at age 6 y, *n*/total (%)^5^		44/817 (5.4)
Overweight children at age 7 y, *n*/total (%)^5^		40/770 (5.2)
Overweight children at age 8 y, *n*/total (%)^5^		59/802 (7.4)

1 Values are mean ± SD or *n* (%) unless otherwise indicated.

2 Complete follow-up: ≥1 outcome measurement between ages 6 and 8 y.

3 Missings excluded.

4 Percentage of valid values (i.e., excluding missing values).

5 Overweight is defined as BMI *z* scores >1.04, corresponding to the 85th percentile.

### Associations with child anthropometric measures

We report the results of the fully adjusted models (**Tables 3**, **4**) because all models for any given outcome yielded generally similar results in terms of the magnitude and direction of the associations.

**TABLE 3:** Associations of maternal plasma choline and betaine concentrations in the third trimester of pregnancy with child birth weight; weight gain in the first year of life; and weight, height, and BMI *z* scores at 1–2 y of age^1^

	Birth weight,^2^ g (*n* = 1331)	Weight gain in first year,^2^ g (*n* = 1208)	Weight *z* score at 1–2 y^3^ (*n* = 1263)	Height *z* score at 1–2 y^3^ (*n* = 1263)	BMI *z* score at 1–2 y^3^ (*n* = 1263)
Choline					
Per µmol/L (95% CI)	−8.0 (−17.0, 0.9)	19.5 (1.1, 38.0)*	0.01 (−0.01, 0.03)	−0.01 (−0.03, 0.01)	0.02 (0.00, 0.04)*
Per SD	−23.2	56.6*	0.03	−0.03	0.06*
*P* value	0.08	0.04*	0.19	0.48	0.02*
Betaine					
Per µmol/L (95% CI)	1.5 (−4.2, 7.1)	12.3 (0.8, 23.9)*	0.01 (−0.00, 0.02)	0.00 (−0.01, 0.01)	0.01 (−0.00, 0.02)
Per SD	6.9	56.6*	0.05	0.01	0.05
*P* value	0.61	0.04*	0.13	0.78	0.08

1 Values are regression coefficients (95% CIs) per 1 µmol/L and per SD of choline and betaine unless otherwise indicated. Analyses were adjusted for child sex, age of outcome measurement (except for birth weight), child ethnicity, design variables (storage time, type of tube used for storage, pregnancy duration at time of blood collection, year and season of blood collection, recruitment group), and maternal covariables (living region within the Netherlands, education, gravidity, age at the beginning of pregnancy, height, prepregnancy BMI, alcohol use in the third trimester, smoking in the third trimester, and gestational diabetes). *Significant associations.

2 Analyzed with multivariable linear regression.

3 Analyzed with generalized estimation equations for repeated measurements with an unstructured correlation structure.

**TABLE 4:** Associations of maternal plasma choline and betaine concentrations in the third trimester of pregnancy with child odds of belonging to the lowest 15 BMI percentiles at 1–2 y and to the highest 15 BMI percentiles at 1–2 and at 6–8 y^1^

	BMI *z* score <−1.04 (15th percentile) at 1–2 y^2^ (*n* = 1263)	BMI *z* score >1.04 (85th percentile) at 1–2 y^2^ (*n* = 1263)	Overweight at 6–8 y^2,3^ (*n* = 1005)	Overweight at 6–8 y (based on home-visits data)^3,4^ (*n* = 648)
Choline				
Per µmol/L (95% CI)	0.97 (0.93, 1.02)	1.08 (1.03, 1.10)*	1.04 (0.98, 1.11)	1.07 (0.96, 1.19)
Per SD	0.93	1.26*	1.13	1.23
*P* value	0.29	<0.001*	0.19	0.20
Betaine				
Per µmol/L (95% CI)	0.99 (0.96, 1.02)	1.03 (1.00, 1.07)*	1.00 (0.95, 1.06)	1.02 (0.94, 1.10)
Per SD	0.94	1.17*	1.02	1.07
*P* value	0.40	0.03*	0.88	0.70

1 Values are ORs per 1 µmol/L and per SD of choline and betaine. Analyses were adjusted for child sex, age of outcome measurement, child ethnicity, design variables (storage time, type of tube used for storage, pregnancy duration at time of blood collection, year and season of blood collection, recruitment group), and maternal covariables (living region within the Netherlands, education, gravidity, age at the beginning of pregnancy, height, prepregnancy BMI, alcohol use in the third trimester, smoking in the third trimester, and gestational diabetes). All growth outcomes were parent-reported unless otherwise specified. *Significant associations.

2 Analyzed with logistic generalized estimation equations.

3 Overweight is defined as BMI *z* scores >1.04, corresponding to the 85th percentile.

4 Analyzed with logistic regression.

#### Birth weight

At birth, maternal third-trimester plasma choline showed a tendency toward a negative association with birth weight (β: −8.0 g; 95% CI: −17.0, 0.9 g; *P* = 0.08), with an effect size of 8 g lower birth weight per 1 µmol/L of plasma choline. Plasma betaine was not associated with birth weight (β: 1.5 g; 95% CI: −4.2, 7.1 g).

#### Growth at 1 and 2 y

Maternal plasma choline and betaine were found to be positively associated with weight gain in the first year of life (β_choline_: 19.5 g; 95% CI: 1.1, 38.0 g and β_betaine_: 12.3 g; 95% CI: 0.8, 23.9 g).

At 1 and 2 y, maternal plasma concentrations of choline and betaine were not associated with child weight and height *z* scores. By contrast, plasma choline was positively associated with BMI *z* score at those ages (β: 0.02; 95% CI: 0.00, 0.04) (Table 3). In addition, higher choline and betaine were both associated with higher odds of having a BMI *z* score > 1.04 (85th percentile) at 1–2 y (OR_choline_: 1.08; 95% CI: 1.03, 1.10 and OR_betaine_: 1.03; 95% CI: 1.00, 1.07), but not with the odds of having a BMI *z* score <−1.04 (15th percentile) (Table 4).

#### Overweight at 6–8 y

At age 6–8 y, maternal plasma choline and betaine were not significantly associated with the risk of the child becoming overweight (BMI *z* score >1.04 based on parent-reported height and weight) (Table 4). These results were confirmed in the subgroup of children in which height and weight were measured by a health nurse during a home visit (Table 4, right column).

#### Sensitivity analyses

Performing analyses without year and season of blood collection, adding the covariable breastfeeding duration, or excluding preterm deliveries or women with diabetes in pregnancy yielded no substantially different results. No statistically significant interactions with breastfeeding duration or folic acid supplementation were found either. We found a statistically significant interaction between betaine and child sex (*P*_interaction_ = 0.03) in the models for overweight between ages 6 and 8 y using home-visits data. Stratified analyses revealed a higher OR of becoming overweight between ages 6 and 8 y in boys (OR: 1.17; 95% CI: 1.02, 1.34) but not in girls (OR: 0.93; 95% CI: 0.84, 1.04) per 1 µmol/L of betaine.

## Discussion

We studied maternal plasma choline and betaine in the third trimester of pregnancy and their associations with child anthropometric measurements from birth up to the age of 8 y. As main results, we found that each 1-µmol/L increase of maternal plasma choline was associated with a mean 20-g higher weight gain in the first year of life. At 1–2 y, choline was associated with higher BMI *z* scores and slightly higher odds of having a BMI *z* score >1.04 (OR: 1.08). Each 1-µmol/L increase of maternal plasma betaine was associated with 12 g higher weight gain in the first year of life, as well as with higher odds of having a BMI *z* score >1.04 at 1 and 2 y (OR: 1.03). Moreover, maternal plasma betaine was associated with higher odds of boys being overweight between ages 6 and 8 y (OR: 1.17) when using home-visits data, but not parent-reported data.

### Comparison with previous studies

There is inconsistent evidence on the association of maternal plasma choline and betaine with child anthropometric measurements. Our results on the lack of associations with birth weight are in agreement with earlier longitudinal studies (12, 18, 19). In contrast, van Lee et al. (17) found a positive association between maternal plasma choline in week 11 and weeks 26–28 of gestation and BMI *z* scores at birth in 2 independent cohorts. However, women with plasma choline in the fifth quintile (mean = 11.7 µmol/L) were older, had higher prepregnancy BMI and gestational weight gain, and more frequent gestational diabetes than women in the lowest quintile (mean = 7.1 µmol/L), suggesting that the associations with the anthropometric measurements of the newborn could be confounded or explained by maternal overweight or insulin resistance. With regard to betaine, our results are in agreement with a previous study that reported no association between betaine and birth weight (18); instead, van Lee et al. (20) and Du et al. (19) reported a negative association, the latter only in boys. In line with our findings at 1–2 y and 6–8 y, van Lee at al. (17) reported maternal choline to be associated with higher BMI *z* scores at 2–3 y, but not with anthropometric measurements at 5 y.

Contradictory results from the literature could be due to considering plasma choline and betaine as surrogate markers of choline intake and/or fetal transfer. The validity of circulating free choline concentrations as a specific indicator of dietary choline exposure is uncertain; although higher-dose supplementation could increase plasma choline, differences in plasma choline in unsupplemented people might not sensitively reflect more subtle dietary intake differences (26). Concentrations of choline increase during pregnancy, mostly because of high estrogen, and those of betaine decline partly owing to the termination of folic acid supplements after the first trimester (27, 28). The associations between maternal choline or betaine at different time points in pregnancy and infant growth could therefore show interactions with nutrients such as folate (25) or hormones that simultaneously affect choline or betaine as well as growth. Nevertheless, we found no effect modification by folic acid supplementation in the present study. Besides, the associations between plasma choline markers and child growth outcomes could also differ in studies including intrauterine growth retardation or compromised maternal vasculature plasticity (e.g., pre-eclampsia).

We have shown that betaine might be associated with growth during the first years of life, and that at late childhood this association might be sex-specific. The possibility that choline and betaine influence males and females differently has been raised before. In adults, choline and betaine concentrations correlated with a lower percentage of body fat in men, whereas serum choline correlated positively with weight, BMI, and waist circumference in women (29). The expression of several enzymes in one-carbon metabolism is sex-dependent and might lead to higher choline and betaine concentrations in females than in males (30). Choline supplementation to pregnant mice was found to have sex- and gestational day–dependent effects on placenta processes (31). We hypothesize that males might be more dependent on choline and betaine exogenous supply (either in utero, through breast milk, or from the child's own diet) than females.

### Evidence on the role of choline in child growth from supplementation studies

Controlled supplementation trials of choline during pregnancy can provide evidence on a potential causal role of choline on child growth. Birth weight and length of the neonates did not differ when women received 480 compared with 930 mg choline/d from week 27 of pregnancy until birth (32). However, a typical European diet may provide between 291 and 374 mg choline/d (33), suggesting that there could be a threshold for choline intake that might influence growth. In this line, animal models have suggested that choline supplementation could be more effective under choline-deficiency conditions (34, 35), and have more pronounced effects on fetal growth in pregnancies with placental insufficiency or compromised fetal growth (36). A study providing 2 g choline/d (compared with placebo) from mid-pregnancy to delivery in heavy-drinking pregnant women showed no difference in birth size between the study groups, but the children from the choline-supplemented mothers showed higher catch-up growth in weight and head circumference at 6.5 and 12 mo (37).

Choline and betaine may interact with fat and glucose metabolism, especially when these macronutrients are in excess. Choline supplementation to pregnant dams fed a high-fat diet (a model of maternal obesity) normalized homeostasis of placental macronutrients (14) and reduced hepatic triglyceride accumulation and the expression of lipogenic genes in the offspring (15). Betaine, in turn, normalized the weight gain of fetuses from dams fed a high-fat diet, decreased body fat percentage, and prevented hepatic triglyceride accumulation similarly to choline (16), although betaine's effects on fetal growth and tissue composition appear to have distinct mechanisms.

### Strengths and limitations

The strengths of the present study include the large sample size, the longitudinal design, and the possibility to analyze child repeated anthropometric data up to the age of 8 y. Moreover, we had information available on maternal folic acid supplementation in pregnancy and on breastfeeding duration, which allowed us to explore possible effect modification. This study has some limitations as well. First, plasma samples were collected in the nonfasting state; it is unclear whether this may have randomly influenced the measurements, and hence hampered the possibility to detect existing associations. Second, parent-reported child growth data may be less precise than nurse-reported data, therefore decreasing the chance to find a significant association or interaction in the main group of children where data were reported by the parents. Third, BMI was used as a proxy for overweight; the meaning of BMI and overweight at very early ages is debatable, and we cannot exclude that choline and betaine could show associations with body composition (fat and lean mass) that are not mirrored by BMI (38). Lastly, we did not include data on children's diet after weaning nor on genetic variants that influence endogenous choline and betaine synthesis capacity.

### Conclusion

Maternal plasma choline and betaine concentrations in the third trimester of pregnancy were associated with a higher weight gain of the child in the first year of age, higher BMI *z* scores at 1–2 y (choline only), and higher odds of a BMI *z* score >85th percentile at 1–2 y. In addition, betaine was associated with higher odds of overweight in 6- to 8-y-old boys. Although the reported associations could be temporary and disappear at older ages, the persistent association that we found of betaine with BMI in boys needs to be confirmed in independent studies. Future studies may address the validity of maternal plasma choline and betaine concentrations as markers of maternal intake and fetal transfer. Research is also warranted to investigate whether pregnancy complications and related risk factors such as maternal overweight modify the associations of choline and betaine with child growth.

## Supplementary Material

nqab177_Supplemental_File

## Data Availability

Data described in the article, code book, and analytic code will be made available upon request pending application and approval.

## Notes

Support for the KOALA Birth Cohort Study until 8 y of follow‐up was provided by the Triodos Foundation, Phoenix Foundation, Raphaël Foundation, Iona Foundation, Foundation for the Advancement of Heilpedagogie, FrieslandCampina, the Dutch Sugar Foundation, the Dutch Ministry of Economic Affairs, The Netherlands Organisation for Health Research and Development (ZonMw project no. 2100.0090), and The Netherlands Asthma Foundation grants 3.2.03.48 and 3.2.07.022, all in the Netherlands; and Procter&Gamble Health, Germany. The sponsors had no role in data analysis, interpretation, or publication of the results.

Supplemental Table 1 is available from the “Supplementary data” link in the online posting of the article and from the same link in the online table of contents at https://academic.oup.com/ajcn/.

Abbreviations used: GEE, generalized estimation equation; KOALA, Kind, Ouders en gezondheid: Aandacht voor Leefstijl en Aanleg; UPLC-MS/MS, ultra-performance liquid chromatography–mass spectrometry.
