# Supplementary material for: Maternal plasma choline and betaine in late pregnancy and child growth up to age 8 years in the KOALA Birth Cohort Study
Source: Am J Clin Nutr. 2021 Jun 10;114(4):1438–46. doi: 10.1093/ajcn/nqab177 (PMC8488875; doi:10.1093/ajcn/nqab177)
Supplement: nqab177_Supplemental_File [file nqab177_supplemental_file.docx]

**Supplemental Table 1. Variables included in the investigated regression models. Either maternal plasma choline or betaine concentrations are used as the exposure variable.**

| Step 1 | Child covariables: sex, age of growth measurement  Design variables: storage time, type of tube used for storage, pregnancy duration at time of blood collection, year and season of blood collection, recruitment group |
| --- | --- |
| Step 2 | Addition of child covariable: ethnicity  Addition of maternal covariables: living region within The Netherlands, education, gravidity, age at the beginning of pregnancy, height, prepregnancy BMI, alcohol use in 3^rd^ trimester, smoking in 3^rd^ trimester |
| Step 3 ^1^ | Addition of maternal covariable: gestational diabetes |
| Sensitivity analyses (applied to fully adjusted model) | - Removal of the covariable year and season of blood collection - Addition of the covariable breastfeeding duration (except for the outcome birth weight) - Exclusion of women with preterm deliveries (<37 wk gestation): 9 women in conventional group, 4 in alternative recruitment group - Exclusion of women with diabetes in pregnancy: 19 in conventional group, 1 in alternative recruitment group - Addition of the interaction term with breastfeeding duration (except for the outcome birth weight) - Addition of the interaction term with child sex - Addition of the interaction term with folic acid supplementation |

^1^ Fully adjusted model
